# Chronic kidney disease and risk factor prevalence in Saint Kitts and Nevis: a cross-sectional study

**DOI:** 10.1186/s12882-016-0424-2

**Published:** 2017-01-05

**Authors:** Deidra C. Crews, Kirk N. Campbell, Yang Liu, Odell Bussue, Ingrid Dawkins, Bessie A. Young

**Affiliations:** 1 Division of Nephrology, Department of Medicine, Johns Hopkins University School of Medicine, 301 Mason F. Lord Drive, Suite 2500, Baltimore, MD 21224 USA; 2 Welch Center for Prevention Epidemiology and Clinical Research, Johns Hopkins Medical Institutions, Baltimore, MD USA; 3 Division of Nephrology, Department of Medicine, Icahn School of Medicine at Mount Sinai, New York, NY USA; 4 Caribbean Health and Education Foundation, Takoma Park, MD USA; 5 Division of Nephrology, Department of Medicine, Seattle Puget Sound Veterans Affairs, Kidney Research Institute, University of Washington at Seattle, Seattle, WA USA

**Keywords:** Chronic kidney disease, Epidemiology, Risk factors, Diabetes, Hypertension

## Abstract

**Background:**

The prevalence of chronic kidney disease (CKD) in St. Kitts and Nevis, islands of the West Indies, is unknown. We sought to determine estimates of CKD and its risk factors (e.g. diabetes, hypertension and obesity) in St. Kitts and Nevis.

**Methods:**

This was a chronic disease screening program. Three community-based locations in St. Kitts and Nevis were included in the program. Participants were adult community residents aged ≥18 years. The main outcome measures were estimated CKD prevalence (by serum creatinine-based estimated glomerular filtration rate (eGFR) and dipstick urine albumin); and estimated prevalence of CKD risk factors (diabetes, hypertension and obesity). Logistic regression was used to determine independent predictors of CKD.

**Results:**

One thousand nine hundred seventy eight persons, from Nevis (*n* = 950) and St. Kitts (*n* = 1028) were screened by the Caribbean Health and Education Foundation. Participants’ mean age was 49 ± 15 years, 65% were female, and 99% were black. Fully, 21.5% had diabetes and 53.1% had hypertension; and 40.3% were obese. Mean estimated eGFR was 98 ml/min/1.73 m^2^ (standard deviation = 30) and 4.7% had an eGFR <60 ml/min/1.73 m^2^, indicating CKD. Age [Odds Ratio (OR) = 1.08, 95% Confidence Interval (CI) 1.05–1.11], hypertension (OR = 2.89, 95% CI 1.18–7.07) and diabetes (OR = 3.12, 95% CI 1.80–5.43) were independent predictors of reduced eGFR in models adjusted for age, gender and obesity status. Of those with urine testing in Nevis (*n* = 929), 13.5% had urine albumin ≥30 mg/dL, and diabetes was an independent predictor of this finding (OR = 2.43, 95% CI 1.53–3.87).

**Conclusions:**

CKD and its risk factors were prevalent among adults in St. Kitts and Nevis. Public policy strategies for prevention and treatment of these conditions may be needed to reduce their associated morbidity, mortality and costs.

## Background

Non-communicable chronic diseases such as stroke, heart disease, diabetes, and hypertension are the leading causes of death in the member countries of the Organization of Eastern Caribbean States (which includes Antigua and Barbuda, Commonwealth of Dominica, Grenada, Montserrat, St. Kitts and Nevis, St. Lucia and St. Vincent and the Grenadines), like that of the wider region, accounting for over half of deaths [1]. Diabetes and hypertension are the two major causes of chronic kidney disease (CKD) and end stage renal disease (ESRD) worldwide [2]. Reports from the Caribbean Renal Registry (CRR) also indicate diabetes and hypertension are the leading causes of CKD and ESRD in the Caribbean [2, 3]. Little is known regarding the prevalence of CKD in the Caribbean, particularly in St. Kitts and Nevis.

The Federation of St. Kitts and Nevis, also known as the Federation of Saint Christopher and Nevis, is a two-island country in the West Indies. The capital city is Basseterre on the larger island of St. Kitts and the smaller island of Nevis lies about 2 miles (3 km) southeast of St. Kitts. Persons residing on St. Kitts (population approximately 45,000) have somewhat greater access to goods and services than those on Nevis (population approximately 12,000), although they share similar sociodemographic compositions.

The Caribbean Health and Education Foundation (CHEF) proposed and conducted a screening program for CKD, diabetes, and hypertension to ascertain the prevalence of CKD and major CKD risk factors in St. Kitts and Nevis. The major objective of CHEF’s Caribbean Kidney Screening and Education Program (CKSEP) was to provide rapid CKD screening among adults who are at risk of developing CKD due to personal and/or family history of diabetes, hypertension or CKD and inform these individuals about CKD, enabling them to better manage their risk. In this manuscript, we report estimates of CKD and its risk factors (e.g. diabetes, hypertension and obesity) as assessed during the CKSEP in St. Kitts and Nevis.

## Methods

### Setting and collaboration

CHEF collaborated with the Nevis Renal Society, the St. Kitts Diabetic Association and The Kidney Trust Foundation to execute the CKSEP, utilizing The Kidney Trust Foundation’s rapid CKD screening program model to assist in identifying people with increased risk of developing CKD and ESRD, and educate/empower them to better manage their illnesses. CKSEP took place on the islands of Nevis and St. Kitts on March 17–18 and March 20–21, 2014, respectively. Three visiting nephrologists, in collaboration with local primary care physicians and a local nephrologist, provided on-site individual consultation to participants at the screenings in Nevis and St. Kitts. The nephrologists and primary care providers were joined by nurses, phlebotomists, medical students and lab technician volunteers. Other volunteers were recruited for registration of individuals, assistance with guiding participants through the screening process, and crowd management.

### Participants

The CKSEP was advertised for several weeks on local radio, television and at community-based clinics as an opportunity for individuals with risk factors for CKD (diabetes, hypertension and/or obesity) to be screened. Adults ≥18 years of age were eligible. Otherwise, there were no exclusions. All participants provided written consent for screening.

### Measurements

Participants were asked sociodemographic questions (age, gender, race/ethnicity), and were asked about their personal (self-report) and family history of diabetes, hypertension and kidney disease, and the name of their regular physician. During the screening, the CKSEP team assessed participants’ height, weight [for body mass index (BMI) (weight/height^2^) calculation], and seated systolic and diastolic blood pressure (mmHg) using automated cuffs. BMI 25 to 29.9 kg/m^2^ was considered overweight, and ≥30 kg/m^2^ was considered obesity. Systolic blood pressure ≥140 mmHg or diastolic blood pressure ≥90 mmHg were considered diagnostic of hypertension.

A venous blood sample for each participant was processed directly on site, using i-STAT Portable Clinical Analyzer (Abbott Laboratories, Abbott Park, IL, USA), which performs rapid blood analysis and displays quantitative test results in approximately 3–5 min. Calibration is automatically performed as part of the iSTAT System test cycle. Quality assurance/performance of the iSTAT System (as performed by The Kidney Trust staff) was verified using the internal or external electronic simulator every 24 h of testing. Serum creatinine was assessed using i-STAT. Estimated glomerular filtration rate (eGFR) was calculated using the 4 variable Modification of Diet in Renal Disease study equation [4] and used to determine stage of CKD [5]. Estimated GFR <60 ml/min/1.73 m^2^ was considered to define reduced eGFR. Random serum glucose was assessed by fingerstick samples using commercially available glucometers (Contour Next and Precision Xtra). Non-fasting fingerstick glucose at or exceeding 200 mg/dL was considered diagnostic of diabetes mellitus [6]. Urine dipsticks were used to assess evidence of albuminuria (Siemens Multistix 10 SG and Siemens CLINITEK Microalbumin 2). Urine albumin results were reported as 0-trace, 30–100 mg/dL, 100–300 mg/dL, and >300 mg/dL. Values 30 and above were considered as evidence of albuminuria. Due to limited availability of urine dipsticks, only high risk participants in Nevis (i.e. those with known diabetes or hypertension) underwent screening for albuminuria.

### Data analysis

Screening data were initially recorded on paper forms and later entered into an electronic database. Data were analyzed individually and together for both St. Kitts and Nevis. Comparisons were also made between islands. Means and proportions for demographic and clinical variables were obtained using t-tests and chi squared tests where appropriate. Serum glucose and blood pressure were described continuously, and as binary variables (yes/no for diabetes or hypertension). Overweight and obesity were each described categorically. Estimated GFR was described continuously and in categories and stages of CKD. Albuminuria was described categorically. Multivariable logistic regression was used to examine the direction, magnitude and independence of the associations of demographic and clinical variables with prevalent reduced eGFR (defined as <60 ml/min per 1.73 m^2^) or albuminuria. In the regression models, age was modeled continuously, while all other covariates were modeled as binary variables.

## Results

A total of 1978 presented to the screening programs on the islands of Nevis and St. Kitts and underwent screening. In Nevis, 950 participants were screened at the Anglican Church Hall in Charlestown. In St. Kitts, 1028 participants were screened at two venues, which operated simultaneously - Newtown and McKnight Community Centers in Basseterre.

### Screening findings in Nevis

The participants screened in Nevis represented approximately 8% of the total population, which is approximately 12,000. The overwhelming majority of participants were English-speaking. Those speaking Spanish or Saint Kitts Creole were able to be accommodated by the language skills of the screening team. The mean age was 49 years, and the majority of participants were female (64%). The overwhelming majority of participants were of black race (Table 1). Many individuals screened had risk factors for CKD, including 28% who self-reported a diagnosis of hypertension and 16.8% who reported having diabetes. Additionally, the average systolic blood pressure of the screened population fell in the ‘stage 1 hypertension’ range [7]. Notably, 8.2% of participants who underwent serum glucose testing had a value exceeding 200 mg/dL, and 12 (17.1%) of these individuals were unaware of a diagnosis of diabetes (based on their answers to our screening questionnaire). The majority of participants were found to be either overweight (38%) or obese (38%). Overweight and obesity were more prevalent among females, with females comprising 62% of overweight and 75% of obese persons screened.

**Table 1:** Participant characteristics and diabetes, hypertension and obesity screening findings among adults in St. Kitts and Nevis, overall and by island

Characteristic/finding	All participants	Results for all	Number of Nevis participants assessed	Nevis results	Number of St. Kitts participants assessed	St. Kitts results	P*
Demographics							
Age in years, mean (SD)	1978	49 (15)	950	49 (15)	1028	49 (15)	0.69
Age category, *n* (%)	1978		950		1028		0.06
< 40 years		541 (27)		252 (26.5)		289 (28.1)	
40–49 years		401 (20)		217 (22.8)		184 (17.9)	
50–59 years		528 (27)		248 (26.1)		280 (27.2)	
≥ 60 years		508 (26)		233 (24.5)		275 (26.8)	
Female gender, *n* (%)	1978	1285 (65)	950	609 (64)	1028	676 (66)	0.44
Race/ethnicity, *n* (%)	1978		950		1028		0.14
Black		1964 (99.3)		939 (98.8)		1025 (99.7)	
White		5 (0.3)		4 (0.4)		1 (0.1)	
Hispanic		9 (0.5)		7 (0.7)		2 (0.2)	
Chronic Kidney Disease Risk Factors							
Systolic blood pressure, mean (SD)	1971	138 (66)	950	140 (63)	1021	136 (68)	0.13
Systolic blood pressure, ≥140 mmHg, *n* (%)	1971	761 (38.6)	950	412 (43.4)	1021	349 (34.2)	<0.001
Diastolic blood pressure, mean (SD)	1964	79 (15)	944	82 (13)	1020	76 (15)	<0.001
Diastolic blood pressure, ≥90 mmHg, *n* (%)	1964	406 (20.7)	944	239 (25.3)	1020	167 (16.4)	<0.001
Self-reported hypertension, *n* (%)	1978	637 (32)	950	267 (28)	1028	370 (36)	<0.001
Self-reported or measured hypertension, *n* (%)	1978	1050 (53.1)	950	527 (56)	1028	523 (50.9)	0.04
Serum glucose in mg/dL, mean (SD)	1518	127 (64)	857	128 (60)	661	125 (69)	0.44
Serum glucose ≥200 mg/dL, *n* (%)	1518	133 (8.8)	857	70 (8.2)	661	63 (9.5)	0.35
Self-reported diabetes mellitus, *n* (%)	1978	403 (20.4)	950	160 (16.8)	1028	243 (23.6)	<0.001
Self-reported or lab-confirmed diabetes mellitus, *n* (%)	1978	426 (21.5)	950	172 (18.1)	1028	254 (24.7)	<0.001
Overweight (BMI 25 to 29.9 kg/m^2^), *n* (%)	1494	529 (35.4)	904	340 (37.6)	590	189 (32.0)	0.03
Obesity (BMI ≥30 kg/m^2^), *n* (%)	1494	602 (40.3)	904	345 (38.2)	590	257 (43.6)	0.04

*Abbreviations*: *SD* standard deviation, *n* number of individual participants, *BMI* body mass index

*P values indicate whether findings on Nevis versus St. Kitts were statistically significantly different

### Screening findings in St. Kitts

The 1028 persons screened in St. Kitts represented approximately 2% of the total population, which is estimated to be 45,000. Similar to Nevis, participants were a mean age of 49 years, the majority were female (66%), and the overwhelming majority were of black race (Table 1). A total of 36% self-reported a diagnosis of hypertension and 23.6% reported having diabetes. Additionally, the average systolic blood pressure of the screened population fell in the ‘pre-hypertension’ range. Fully, 9.5% of participants who underwent serum glucose testing had a value that exceeded 200 mg/dL, and 11 (17.5%) of these individuals were unaware of a diagnosis of diabetes. The majority of participants assessed (*n* = 590) were found to be either overweight (32%) or obese (44%). Overweight and obesity were more prevalent among females, with females comprising 56% of overweight and 76% obese persons screened.

### Kidney disease in Nevis and St. Kitts

Among the kidney disease measures administered, persons were found to have a mean eGFR of 98 mL/min/1.73 m^2^ (SD) and 4.7% had an eGFR <60 mL/min/1.73 m^2^. Nevisians had a lower average eGFR than Kittitians screened, but there were no differences in the prevalence of eGFR < 60 ml/min per 1.73 m^2^. The majority of participants (88%) with an eGFR < 60 mL/min/1.73 m^2^ would be considered to have CKD stage 3 by National Kidney Foundation guidelines if confirmed on repeat analyses [5]. Eleven persons had more advanced CKD (eGFR <30/CKD stages 4 and 5). A total of 929 Nevisians underwent urine testing, and 126 individuals (13.5%) had a urine albumin level of at least 30 mg/dL, which is suggestive of kidney damage. Five persons screened (0.5%) had significantly elevated urine albumin (of at least 300 mg/dL) (Table 2). Due to a limited supply of dipsticks, only 183 participants underwent screening for urine albumin in St. Kitts. These were primarily persons with risk factors for CKD (e.g. diabetes), and/or those identified to have reduced eGFR during screening. Among them, 141 individuals had a urine albumin of at least 30 mg/dL. Eleven persons screened in St. Kitts had significantly elevated urine albumin (of at least 300 mg/dL).

**Table 2:** Kidney disease screening findings among adults in St. Kitts and Nevis, overall and by island

Characteristic/Finding	All participants *N*	Results for all	Number of Nevis participants assessed	Nevis results	Number of St. Kitts participants assessed	St. Kitts results	P*
Kidney Disease Measures							
Serum creatinine in mg/dL, mean (SD)	1978	0.9 (0.3)	950	0.9 (0.3)	1028	0.9 (0.4)	0.04
Estimated GFR (mL/min/1.73 m^2^) mean (SD)	1978	98 (30)	950	95 (30)	1028	100 (29)	<0.001
Estimated GFR <60 mL/min/1.73 m^2^, *n* (%)	1978	93 (4.7)	950	44 (4.6)	1028	49 (4.8)	0.89
Estimated GFR <45 mL/min/1.73 m^2^, *n* (%)	1978	32 (1.6)	950	18 (1.9)	1028	14 (1.4)	0.35
CKD stage, *n* (%) [among persons with estimated GFR < 60]	93	--	--	--	--	--	0.87
-Stage 3 (estimated GFR 30 to 59)		82 (88.2)	--	38 (86.4)	--	44 (90)	
-Stage 4 (estimated GFR 15–29)		9 (9.7)	--	5 (11.4)	--	4 (8)	
-Stage 5 (estimated GFR <15)		2 (2.2)	--	1 (2.3)	--	1 (2)	
Urine protein, *n* (%)	--	--	929				
0 to trace mg/dL		--		803 (86.4)			
≥ 30 to <100 mg/dL		--		103 (11.1)			
≥ 100 to <300 mg/dL		--		18 (1.9)			
≥ 300 mg/dL		--		5 (0.5)			
Urine protein ≥30 mg/dL, *n* (%)	--	--	929	126 (13.5)			

*Abbreviations*: *SD* standard deviation, *n* number of individual participants, *GFR* glomerular filtration rate, *BMI* body mass index

*P values indicate whether findings on Nevis versus St. Kitts were statistically significantly different

Independent predictors of eGFR < 60 ml/min per 1.73 m^2^ in the overall population (both islands), included age, self-reported and/or measured hypertension and self-reported and/or lab-confirmed diabetes mellitus (Table 3). Similar predictors were observed for both St. Kitts and Nevis, however, hypertension was not a statistically significant predictor of reduced eGFR in St. Kitts following adjustment for obesity. Diabetes (self-reported and/or lab-confirmed) was the only observed independent predictor of albuminuria in Nevis.

**Table 3:** Multivariable logistic regression models of predictors of kidney disease measures among adults in St. Kitts and Nevis

Outcome	*N* for analysis	Variables	Odds ratio	95% Confidence interval
Nevis and St. Kitts Combined				
Estimated GFR < 60 mL/min/1.73 m^2^	1978	Age, in years	1.08	1.05–1.11
		Male vs. female gender	1.27	0.73–2.22
		Hypertension vs. no hypertension	2.89	1.18–7.07
		Diabetes vs. no diabetes	3.12	1.80–5.43
		Obesity (BMI ≥ 30) vs. no obesity	1.30	0.72–2.35
Nevis				
Estimated GFR < 60 mL/min/1.73 m^2^	950	Age, in years	1.07	1.04–1.10
		Male vs. female gender	1.08	0.54–2.15
		Hypertension vs. no hypertension	3.73	1.08–12.86
		Diabetes vs. no diabetes	2.93	1.48–5.79
		Obesity (BMI ≥ 30) vs. no obesity	1.32	0.64–2.73
Albuminuria (≥30 mg/dL)	929	Age, in years	1.00	0.99–1.02
		Male vs. female gender	1.21	0.81–1.83
		Hypertension vs. no hypertension	1.34	0.84–2.15
		Diabetes vs. no diabetes	2.43	1.53–3.87
		Obesity (BMI ≥ 30) vs. no obesity	1.06	0.70–1.62
St. Kitts^a^				
Estimated GFR < 60 mL/min/1.73 m^2^	1028	Age, in years	1.12	1.05–1.19
		Male vs. female gender	1.57	0.59–4.17
		Hypertension vs. no hypertension	1.99	0.53–7.49
		Diabetes vs. no diabetes	3.62	1.38–9.54
		Obesity (BMI ≥ 30) vs. no obesity	0.69	0.49–3.66

Note: All variables were simultaneously included in the models presented and were modeled as detailed

*Abbreviations*: *BMI* body mass index

^a^Predictors of proteinuria are not included for St. Kitts given the limited number of participants who underwent testing (183), and their being selected for screening due to increased risk of proteinuria

## Discussion

In the context of a screening program, we found that CKD and its risk factors were prevalent among adults in St. Kitts and Nevis, including 4.7% with reduced kidney function and (in Nevis) 13.5% with albuminuria. Diabetes, hypertension and obesity were highly prevalent in this population, suggesting opportunities for CKD prevention through early detection and evidence-based treatment of these chronic conditions.

There is limited information available from the Caribbean Renal Registry regarding CKD prevalence in the English-speaking Caribbean. These data were not derived from population-based screens, but rather were documented by some participating providers of care delivered to their specific patients. For example, in Jamaica there are a reported 968 patients (population 2.8 million) with CKD, which includes 576 patients on dialysis [8]. Trinidad and Tobago (population 1.2 million) reported 564 patients on dialysis. Diabetes and hypertension are the main causes of ESRD in the English-speaking Caribbean as in the US. In Trinidad and Tobago, Antigua and Barbuda and the British Virgin Islands, diabetes is the primary cause of CKD (30.5, 44 and 56% respectively) with hypertension second (16.5, 23.3 and 28% respectively) [3]. In Jamaica and St. Lucia hypertension is the most common etiology of CKD (35.2 and 53.3% respectively) followed by diabetes (29.7 and 25.5% respectively) [3]. The high prevalence of diabetes and hypertension in St. Kitts/Nevis therefore increases the risk of CKD in this population.

We detected a greater self-reported diabetes prevalence than previously documented by the Pan American Health Organization for St. Kitts/Nevis (8%) [2]. The World Health Organization (WHO) also reported a greater than average regional diabetes prevalence among women in St. Kitts/Nevis [3]. The mean prevalence of diabetes in the WHO Americas region is 11.5% for men and 9.9% for women [3]. According to the WHO, 14.6% of men and 13.6% of women in St. Kitts/Nevis have diabetes [3]. It should be noted that the WHO estimates prevalence based on an elevated fasting glucose or use of medication for diabetes. The high self-reported prevalence in our sample should therefore be interpreted with caution, particularly since one screening site in St. Kitts was in close proximity to a diabetes care center. The self-reported prevalence of diabetes in St. Kitts was 23.6%. Still, the Nevis screening center was not close to such a facility and the self-reported diabetes prevalence was still elevated at 16.8%. Our ability to diagnose diabetes was limited since we were not able to measure fasting glucose or hemoglobin A1C. However, the high proportion (8.7%) found to have a random serum glucose ≥ 200 mg/dl, may provide an estimate of the level of blood glucose control in the population.

The 2008 WHO data for St. Kitts/Nevis reported 40.7% for obesity and 37.4% for hypertension [9]. These values were consistent with our screening findings. It should be noted that proportion measured to be obese in our screening program as well as the WHO data reflect a significantly greater mean than the regional Americas average (23.5% for males and 29.7% for females) [10]. The proportion with hypertension we detected is consistent with regional estimates that reflect a significantly higher disease burden in individuals of African descent. In the US, the age-adjusted prevalence of hypertension overall is 29.1%, while for black adults it is 42% [11].

There were limitations of our screening program that deserve consideration. First, we included adults who presented for screening and targeted the advertisements about our program to high-risk individuals. Further, we did not conduct population-based sampling of participants, or have an available reference point (i.e. known age/gender distribution of CKD) to which to compare our participants. Thus, we may have detected a higher proportion of individuals with CKD and its risk factors than would be obtained in a population-based sample. Second, we did not standardize the measurement of blood pressure, and therefore, measurement error was possible. Third, we were limited in our ability to administer urine albumin tests to all participants, and therefore preferentially did so among higher risk participants, which make our albuminuria findings only generalizable to individuals with CKD risk factors.

## Conclusions

To our knowledge this is the first report of a screening activity of this magnitude for CKD in the Caribbean. This first effort lays the ground work for developing health strategies for prevention and treatment of CKD and ESRD and their risk factors in St. Kitts and Nevis, diseases of which are associated with high cost, morbidity and mortality. Development of public health strategies to improve access to primary care, treatment of conditions such as diabetes and hypertension, increasing public health awareness of obesity and increasing awareness of the benefits of lifestyle changes, as well as education of primary care providers as to how to assess, treat, and intervene on their patients for these most prevalent causes of CKD/ESRD, are specific recommendations that may help to eventually decrease the incidence of CKD and ESRD in St. Kitts and Nevis.

## Abbreviations

CHEF: Caribbean Health and Education Foundation
CI: Confidence interval
CKD: Chronic kidney disease
CKSEP: Caribbean Kidney Screening and Education Program
CME: Continuing medical education
CRR: Caribbean Renal Registry
EGFR: Estimated glomerular filtration rate
ESRD: End stage renal disease
IRB: Institutional Review Board
NIDDK: National Institute for Diabetes and Digestive and Kidney Diseases
OR: Odds ratio
WHO: World Health Organization
